# Analysis of inflammatory parameters and disease severity for 88 hospitalized COVID-19 patients in Wuhan, China

**DOI:** 10.7150/ijms.47935

**Published:** 2020-07-25

**Authors:** Xia Xu, Mu-Qing Yu, Qian Shen, Lian-Zhong Wang, Rong-Di Yan, Meng-Yu Zhang, Jian-Yu Liu, Yi-Qing Qu

**Affiliations:** 1 Department of Geriatric Medicine, Qilu Hospital of Shandong University.; 2 Department of Pulmonary and Critical Care Medicine, Tongji Hospital, Tongji Medical College, Huazhong University of Science and Technology.; 3 Department of Oncology, Tongji Hospital, Tongji Medical College, Huazhong University of Science and Technology.; 4 Department of Pulmonary and Critical Care Medicine, The Second Affiliated Hospital of Shandong University of TCM.; 5 Department of Pulmonary and Critical Care Medicine, Qilu Hospital, Cheeloo College of Medicine, Shandong University, Jinan 250012, China.; 6 Department of Pulmonary and Critical Care Medicine, Qilu Hospital of Shandong University.

**Keywords:** COVID-19, outbreak phage, clinical characteristics, inflammatory cytokines, disease severity

## Abstract

**Background and aim:** The outbreak of coronavirus disease 2019 (COVID-19) is quickly turning into a pandemic. We aimed to further clarify the clinical characteristics and the relationship between these features and disease severity.

**Methods:** In this retrospective single-center study, demographic, clinical and laboratory data were collected and analyzed among moderate, severe and critically ill group patients.

**Results:** 88 hospitalization patients confirmed COVID-19 were enrolled in this study. The average age of the patients was 57.11 years (SD, ±15.39). Of these 88 patients, the median body mass index (BMI) was 24.03 (IQR, 21.64-26.61; range 15.05-32.39), the median duration from disease onset to hospital admission were 11 days (IQR, 6.50-14.50). 46.59% patients had one or more comorbidities, with hypertension being the most common (26.14%), followed by diabetes mellitus (12.50%) and coronary atherosclerotic heart disease (CAD) (7.95%). Common symptoms at onset of disease were fever (71.59%), cough (59.09%), dyspnea (38.64%) and fatigue (29.55%). 88 patients were divided into moderate (47 [53.41%]), severe (32 [36.36%]) and critically ill (9 [10.23%]) groups. Compared with severe and moderate patients, lymphocytopenia occurred in 85.71% critically ill patients, and serum IL-2R, IL-6, IL-8, TNF-α, LDH, and cTnI were also increased in 71.42%, 83.33%, 57.14%, 71.43%, 100% and 42.86% in critically ill patients. Through our analysis, the age, comorbidities, lymphocyte count, eosinophil count, ferritin, CRP, LDH, PT and inflammatory cytokines were statistically significant along with the disease severity.

**Conclusion:** We found some clinical characteristic and inflammatory cytokines could reveal the severity of COVID-19 during the outbreak phage. Our research could assist the clinicians recognize severe and critically ill patients timely and focus on the expectant treatment for each patient.

## Introduction

In December 2019, Coronavirus Disease 2019 (COVID-19), a fatal zoonotic disease, occurred in Wuhan, Hubei Province, China. The disease which was caused by 2019 novel coronavirus (2019-nCoV) has rapidly spread. Up to March 8, 2020, a total of 80735 COVID-19 cases in China had been confirmed. The pathogen could cause severe respiratory syndrome, including fever, dyspnea, and cough 1-7, along with other systematic damage like acute cardiac or kidney injury 8. The transmission from person to person, caused by a propagated source, was the primary liability to the rapidly outbreak in China even the worldwide 9.

At the early phase of COVID-19, researches indicated that COVID-19 was similar with severe acute respiratory syndrome (SARS) and Middle East respiratory syndrome (MERS) in epidemiology, clinical features, and diagnosis 6,7. Primitive case reports revealed the common symptoms were fever, cough, and myalgia or fatigue, which were similar with normal influenza 10. They estimated the severity of COVID-19 by whether patients living in intensive care unit (ICU), which may neglect some severe patients, and the relationship between disease characteristic, several inflammatory cytokines and disease severity was still not quite clear.

As time went by, COVID-19 turned to outbreak very fast. As of February 4, 2020, the new growth speed of diagnosis rates had reached 20% in China, especially in Hubei Province, which was more serious than any other areas, and the number of growth speed reached 35% (all the data were collected from the official website of National Health Commission of the People's Republic of China). Diversities among different periods of COVID-19 had been reported 11. Furthermore, a newly published meta-analysis showed that, lymphopenia, decreased albumin, increased level of C-reactive protein (CRP) and Erythrocyte sedimentation rate (ESR), as well as Lactate dehydrogenase (LDH), seemed to be the most common abnormal laboratory findings 8. The clinical features of the disease in outbreak phage were still not clear, so we did a descriptive research to state the clinical characteristics and inflammatory indexes of the outbreaking COVID-19.

In this study, we aimed to describe the disease characteristics and figure out the relationship between the disease severity, clinical characteristic and inflammatory cytokines.

## Materials and Methods

### Patients

Our institutional review board approved this retrospective study (KYLL-2020-104). From February 3, 2020, to March 20, 2020, we conducted a retrospective study focusing on the clinical characteristics of confirmed cases of COVID-19 in Tongji Hospital, Tongji Medical College, Huazhong University of Science and Technology, during which the rapid growth speed of diagnosis rated up to 58% in Wuhan, Hubei province. Case definitions of confirmed human infection were in accordance with the COVID-19 Guidelines (trial version 7) from the National Health Committee of the People's Republic of China. COVID-19 patients were diagnosed through the symptoms and computed tomography (CT) image combining the detection of 2019-nCoV RNA or the specific virus IgM and IgG antibody to COVID-19.

We collected data of 88 patients admitted to our hospital with confirmed COVID-19 in Wuhan, Hubei province and extracted the medical records of patients in Tongji Hospital. The data included demographic data, exposure history, medical histories, symptoms, signs, laboratory findings and chest CT image. The severity classifying criteria was based on a previous study 12. In brief, the patients were classified by typical symptoms, radiology manifestation, respiratory rates, pulse oxygen saturation, oxygenation index and existing of respiratory or circulatory failure. A team of doctors who had been treating patients with COVID-19 collected and reviewed the data. Because of the urgent need to collect data on this emerging novel pathogen, the written informed consent was waived. We used a standardized case report form to retrospectively collect the clinical and laboratory data.

### Laboratory tests

All the respiratory and blood specimens were collected at admission. Nasopharyngeal swab specimens collected from all patients were tested by real time polymerase chain reaction (RT-PCR) for 2019-nCoV RNA. And the serum was collected to detect the specific IgM and IgG antibody to COVID-19. Other laboratory tests included complete blood cell count, hepatorenal function (alanine aminotransferase [ALT], aspartate aminotransferase [AST], globulin, LDH, creatinine and urea nitrogen), electrolytes, coagulation profile, myocardial enzyme and inflammatory cytokines (CRP, ferritin, Interleukin [IL]-1β, IL-2R, IL-6, IL-8, IL-10, Tumor Necrosis Factor [TNF]-α). All the detection was finished in the key laboratory of Tongji Hospital.

### Statistical analysis

We summarized continuous variables as either the mean, standard deviation (SD) or median with interquartile range (IQR). For categorical variables, we calculated the percentages of patients in each category. Comparisons between different groups were made using Student t test or Mann-Whitney U test for continuous data, and the χ^2^ or Fisher exact test for categorical data. Correlation coefficients were then calculated between clinical and laboratory findings using Spearman or Pearson correlation as appropriate. All statistical analysis procedures were conducted using SPSS 22.0 software (IBM, Armonk, NY), *p* < 0.05 was considered statistically significant.

## Results

### Clinical characteristics and laboratory parameters

The study population included 88 confirmed and hospitalized COVID-19 patients. The results showed that average age was 57.11 years (SD, ±15.39), and 36 (40.91%) were men. 32 (36.36%) patients were old people (≥ 65 years). The median body mass index (BMI) was 24.03 (IQR, 21.64-26.61; range 15.05-32.39). 10 (11.36%) patients had definite exposure history and 3 of these patients were clustered. Of these 88 patients, 68 (77.27%) of them were tested with positive viral nucleic acid test and 20 (22.73%) of them with specific virus IgM and IgG antibody to COVID-19. The median duration from disease onset to hospital admissions were 11 days (IQR, 6.50-14.50). Among these patients, 41 (46.59%) had 1 or more existing comorbidity, including hypertension (23 [26.14%]), diabetes mellitus (DM) (11 [12.50%]), coronary atherosclerotic heart disease (CAD) (7 [7.95%]), chronic obstructive pulmonary disease (COPD) (4 [4.55%]), and malignancy (4 [4.55%]). The most common manifestations of COVID-19, at the onset of the disease, were fever (63 [71.59%]), cough (52 [59.09%]), dyspnea (34 [38.64%]), fatigue (26 [29.55%]) and diarrhea (22 [25.00%]). Additional symptoms included myalgia, chill, headache, expectoration, vomiting, haemoptysis and pharyngalgia (**Table 1**).

The blood counts of 8 of the 88 (9.09%) patients showed leucopenia (white blood cell count (WBC) <4×10^9^/L), 44 (50.00%) showed lymphocytopenia (lymphocyte count <1.10×10^9^/L), 44 (50.00%) showed eosinoponia (eosinophil count <0.02×10⁹/L) and 16 (18.18%) showed mononucleosis (monocyte count >0.60×10^9^/L). As for inflammatory cytokines, the levels of serum CRP and ferritin increased in 59 (67.05%) and 43 (48.86%) patients, 5 (5.68%) patients had higher serum levels of IL-1β (>5.0 pg/mL). 9 (10.23%) patients had increased serum levels of IL-2R (>710 U/ml). There are 15 (17.05%), 6 (6.82%) and 4 (4.55%) patients with higher levels of serum IL-6 (>7.0 pg/ml), IL-8 (>62 pg/ml) and IL-10 (>9.1pg/ml). Besides, levels of serum TNF-α increased in 11 (12.50%) patients. For liver function parameters, levels of ALT and AST increased in 29 (32.95%) and 32 (36.36%) patients, levels of globulin and LDH increased in 19 (21.59%) and 48 (54.55%) patients. The levels of D-dimer and prothrombin time (PT) increased in 40 (45.45%) and 26 (29.55%) patients. The results indicated that there were 29 (36.36%) patients occurring liver damage, 7 (7.95%) patients occurring myocardial damage which was diagnosed by serum cardiac troponin I (cTnI) elevation (>15.6 μg/L), and 2 patients (2.27%) occurring acute kidney injury in our study with the serum creatinine above 104 μmol/L (**Table 2**).

### The relationship between clinical characteristics and disease severity

As shown in **Table 3**, we divided 88 hospitalized patients into moderate (47 [53.41%]), severe (32 [36.36%]) and critically ill (9 [10.23%]) groups according to the classifying criteria. Interestingly, men were the majority (7 [77.78%]) of the critically ill group patients, while women (52 [59.09%]) seemed slightly more in overall patients. It deserved to be mentioned that the average age was ascending along with the increasing disease severity (52.49 years vs 59.94 years vs 74.78 years, *p<*0.001). BMI seemed to decrease along with the increasing disease severity, the median BMI of moderate, severe and critically ill group were 24.22 (IQR, 21.75-25.89), 24.03 (IQR, 22.04-27.35) and 21.88 (IQR, 16.24-25.45), while it is non-significant statistically (*p=*0.340). A total of 41 (46.59%) patients existed comorbidities. There were 17 (36.17%) patients with comorbidities in the moderate group (n=47), in the severe group (n=32) the number is 17 (53.13%), and there were 7 (77.78%) patients with comorbidities in critically ill group (n=9), which demonstrated the increasing rates of comorbidities in severe and critically ill patients, and there was a dominant p value (*p=*0.047) about comorbidity occurrence among these three groups. Concerning the clinical symptoms, we found there was no dominating diversity between different severities of COVID-19.

### The relationship between laboratory parameters and disease severity

To determine the major differences of clinical features among different group patients, we explored the difference of laboratory parameters according to disease severity (**Table 4**).

The results showed that lymphocyte count (1.34 vs 0.89 vs 0.57, *p<*0.001), eosinophil count (0.07 vs 0.00 vs 0.00, *p=*0.002), CRP (7.20 vs 33.20 vs 98.60, *p<*0.001), LDH (201.5 vs 297.0 vs 493.0, p < 0.001) and PT (13.65 vs 14.20 vs 15.80, *p=*0.019) were statistically significant among the three groups. Moreover, inflammatory cytokines obviously changed among these patients, including ferritin (348.5 vs 504.0 vs 1853.0, *p=*0.002), IL-2R (297.0 vs 478.0 vs 968.0, *p=*0.005), IL-6 (1.88 vs 2.96 vs 34.01, *p=*0.002), IL-8 (7.10 vs 9.25 vs 76.10, *p=*0.003) and TNF-α (6.80 vs 6.60 vs 11.50 *p=*0.008). Patients in critically ill group were more likely to have lymphocytopenia (85.71% vs 68.75% vs 34.04%, *p=*0.002) and eosinoponia (31.91% vs 75.00% vs 83.33%, *p=*0.002), and were more likely to have higher IL-2R (71.43% vs 11.11% vs 6.90%, *p<*0.001), IL-6 (83.33% vs 27.78% vs 17.24%, *p=*0.005), IL-8 (57.14% vs 5.56% vs 3.45%, *p<*0.001), IL-10 (33.33% vs 5.56% vs 3.70%, *p=*0.046), TNF-α (71.43% vs 35.29% vs 0, *p=*0.008), LDH (100% vs 75.00% vs 36.96%, *p<*0.001) and cTnI (42.86% vs 9.52% vs 6.45%, *p=*0.025). The WBC count, monocyte count, globulin, and ALT showed no difference among these three groups. Though the changing levels among three groups seemed non-significant statistically, we still noticed that some parameters increasing apparently in the severe or critically ill group patients especially, such as IL-10 (*p=*0.046) and D-dimer (*p=*0.028), of which the median value and IQR were non-significant but the distribution is disparate.

### The relationship between clinical characteristics and inflammatory cytokines

**Figure 1:**
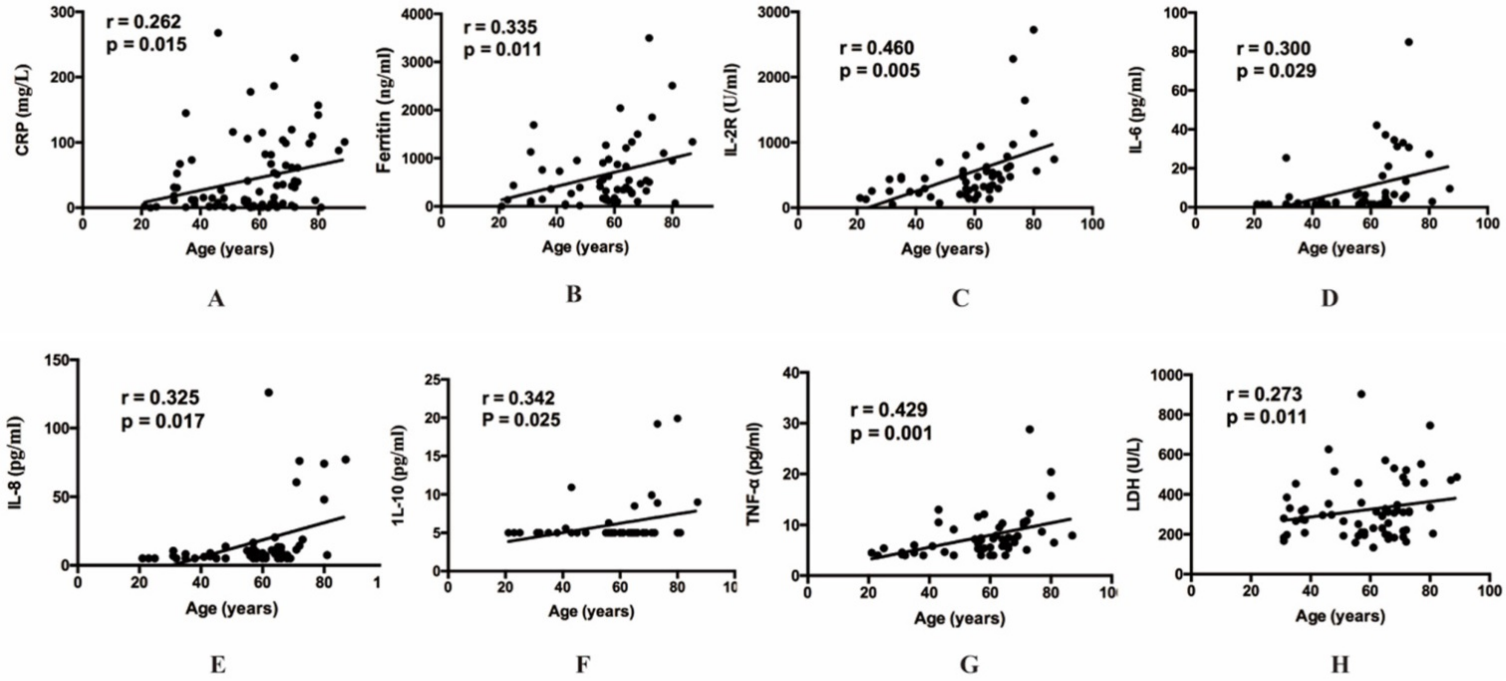
** The relationship between age and inflammatory cytokines.** (**A**) The relationship between age and CRP. (**B**) The relationship between age and ferritin. (**C**) The relationship between age and IL-2R. (**D**) The relationship between age and IL-6. (**E**) The relationship between age and IL-8. (**F**) The relationship between age and IL-10. (**G**) The relationship between age and TNF-α. (**H**) The relationship between age and LDH. CRP, C-reactive protein. IL, Interleukin. TNF, Tumor Necrosis Factor. LDH, Lactate dehydrogenase.

**Figure 2:**
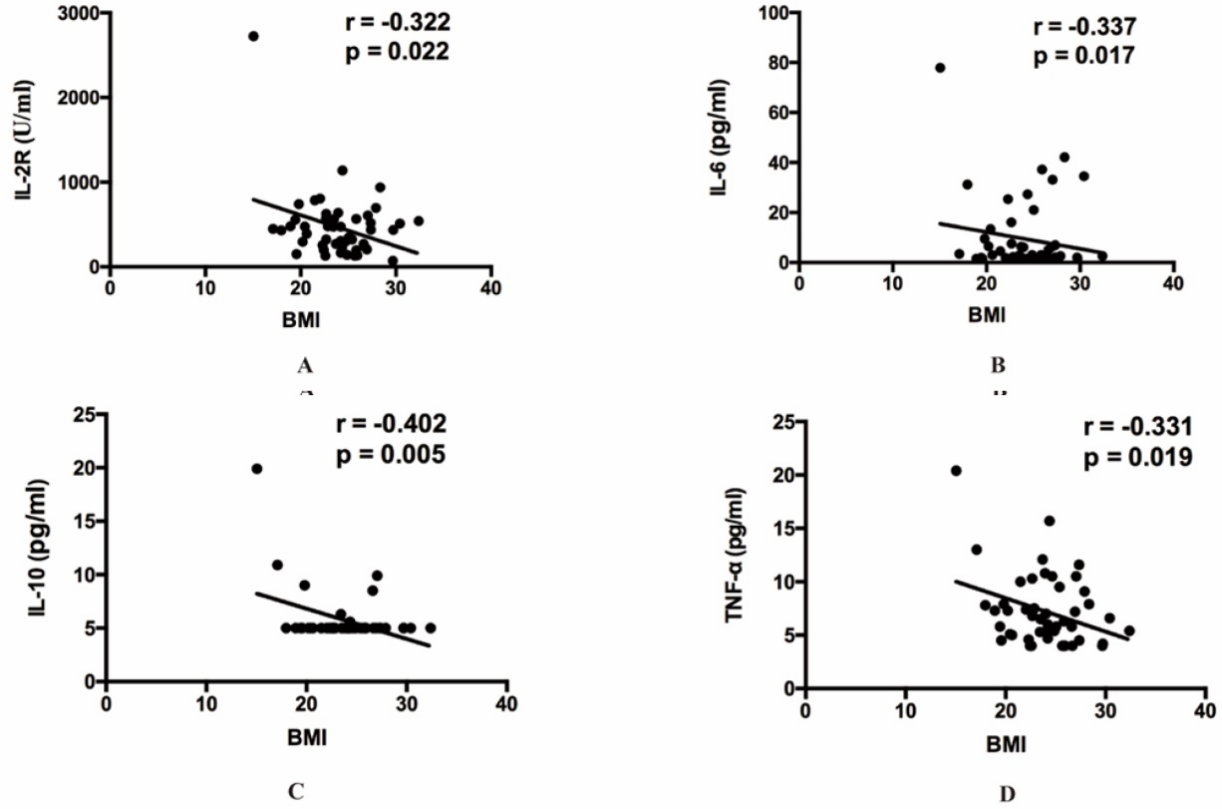
** The relationship between BMI and inflammatory cytokines.** (**A**) The relationship between BMI and IL-2R. (**B**) The relationship between BMI and IL-6. (**C**) The relationship between BMI and IL-10. (**D**) The relationship between BMI and TNF-α. IL, Interleukin. TNF, Tumor Necrosis Factor.

Then our group made correlation analysis to illuminate the relations between inflammatory indexes and other clinical parameters. Among all the inflammatory indexes, CRP (*r=*0.262, *p=*0.015), LDH (*r=*0.273, *p=*0.011), ferritin (*r=*0.335, *p=*0.011), IL-2R (*r=*0.460, *p=*0.005), IL-6 (*r=*0.300, *p=*0.029), IL-8 (*r=*0.325, *p=*0.017), IL-10 (*r=*0.342, *p=*0.025) and TNF-α (*r=*0.429, *p=*0.001) were positively correlated with age (**Figure 1**). The result also indicated that BMI showed positive correlation with IL-2R (*r=*-0.322, *p=*0.022), IL-6 (*r=*-0.337, *p=*0.017), IL-10 (*r=*-0.402, *p=*0.005) and TNF-α (*r=*-0.331, *p=*0.019) (**Figure 2**). Furthermore, to comprehend whether there were relations between different symptoms and inflammatory indexes, our group compared each symptom to the indexes. Then we found decreasing lymphocyte count (0.84 vs 1.21, *p=*0.008), and increasing CRP (34.50 vs 10.90, *p=*0.036), IL-6 (6.67 vs 2.04, *p=*0.015), TNF-α (7.90 vs 5.80, *p=*0.036), PT (14.50 vs 13.70, *p<*0.001) occurred frequently in the patients with dyspnea. And the patients with fever showed decreasing lymphocyte count (0.93 vs 1.33, *p=*0.049), eosinophil count (0.01 vs 0.07, *p=*0.021), and increasing CRP (28.85 vs 5.40, *p=*0.031) and IL-8 (9.90 vs 7.10, *p=*0.045) (**Table 5**).

### Prognostic factors in COVID-19 patients

Our data collection was up to March 20. All the 47 patients in the mild ill group were discharged. In the severe ill group, there was 1 patient turned into critical patient and admitted to intensive care unit with mechanical ventilation, 3 patients were still in the hospital for treatment and the other 28 patients left hospital. In the critically ill group, 2 patients left hospital, 2 patients were still alive with mechanical ventilation, and 4 patients died. The median duration of 2019-nCoV nucleic acid was 22.00 days (IQR, 16.00-27.25). The ICU- and death patients were usually deemed to be caused by acute exacerbation of COVID-19, and there were 10 patients in our study that suffered from COVID-19 exacerbation. To elucidate what influenced the progression of the disease exacerbation, we made a one-way analysis of variance and discovered that gender, age, disease severity, underlying diseases and several laboratory parameters (AST, LDH, IL-6, IL-2R, IL-8, D-dimer and cTnI) affected COVID-19 progress statically significantly (**Table 6**).

## Discussion

Our study was a descriptive research of 88 COVID-19 hospitalized patients in Wuhan, China. As mentioned above, the study was designed to demonstrate the clinical features of COVID-19 in the outbreak phage comparing to the previous data in initiate phage, and found the latent connections between considerable baseline information or laboratory indicators and the severity of the disease. Then we found several hints to the severity of COVID-19 so as to assist the clinicians to recognize severe patients timely and focus on the expectant treatment for each patient.

The epidemiologic characteristic was not definitely in our research, because the patients were collected in the outbreak stage of COVID-19 and the disease spread rapidly. Amounts of patients were infected in daily life and public environment, such as market or restaurant, which led to the simultaneously outbreak and unclearly exposure history.

The median age of 88 patients was similar with the previous reports 13. In spite of the more women in our study, the critically ill group was still mainly constituted of old males, and the result may suggest the old males more easily developed to critically ill patients 14-17. In particular, the analysis showed the age was positively correlated with some indexes, such as CRP, ferritin, LDH, IL-2R, IL-6, IL-8, IL-10 and TNF-α, which may further elucidate that the severe inflammatory response more possibly take place among the old population. The BMI was also observed to decrease in critically ill group, although the p value was not satisfactory which may be caused by the insufficient sample capacity. It needed more evidence to elucidate whether the relatively low BMI makes the disease more severe.

According to the reports at the initiate phage of COVID-19, 64 (46.4%) had 1 or more comorbidities of 138 patients, and was similar with 46.59% in our study. Wang reported the rate (72.2%) of comorbidity in the ICU patients 18, and more than half of the severe and critically ill patients (53.13% and 77.78%) also had the comorbidity in our study, which all revealed that coexisting conditions would intensify the severity of COVID-19.

In consistent with recent studies 6,10,19-20, fever (71.59%) and cough (59.09%) were also the most common symptoms. But among these 88 patients, 22 (25.00%) were suffered with diarrhea which was much more than the previous data (3.80%). Given to the feature that there were no mild patients in our ward, we supposed whether gastrointestinal symptom was correlated with severity of COVID-19, or because the patient waited a long time (11 days) for hospital admission in outbreak phase and they took some medicines outside the hospital.

In accordance with the research published on January 24, 2020, Huang and his colleague found the cytokines and chemokines were elevated in the COVID-19 patients, and made the comparison between ICU and non-ICU patients, which revealed the higher IL-2, IL-7, IL-10, TNF-α, GCSF, IP10, MCP1, MCP1A levels in the ICU patients. Then they proposed that the cytokine storm was associated with disease severity, and indicated the cell response of T-helper-1 (Th1) and T-helper-2 (Th2) cells were both activated 10. We also discovered there were higher levels and proportion of the elevation of LDH, IL-2R, IL-6, IL-8, IL-10 and TNF-α, furthermore, there were only higher levels of CRP and ferritin in the critically ill COVID-19 patients. By contrast, the identical phenomenon likewise occurred in both Severe Acute Respiratory Syndrome Coronavirus (SARS-CoV) and Middle East Respiratory Syndrome Coronavirus (MERS-CoV) infection 21,22, which was associated with pulmonary inflammation and extensive lung damage.

In our present study, there are still some notable limitations. Some cases had incomplete information of the exposure history, and the laboratory parameters in view of objective lack of infrastructure and subjective wishes of patients. And the statistical bias may influence our results because of the constricted sample capacity. There is another limitation also should be addressed is that children not been evaluated, which because children are not hospitalized in our ward. These would have been particularly useful to understand the difference of inflammatory cytokines between children and adults, since major studies reported that children have in general a milder disease with rare exception 23.

In conclusion, our results indicated that there were 29 (32.95%) patients occurring liver damage, this may be caused by the direct attack of COVID-19 virus 24 or by use of drugs, which would affect the liver function 25. Through the comparison between symptoms and inflammatory indexes, we found that dyspnea and fever were correlated with some indexes, such as lymphocyte count, CRP and inflammatory cytokines, which could suggest us to take measures in these patients with dyspnea or fever at the early stage of COVID-19 and that these patients symptom severity might be more severe than those without dyspnea or fever.

## Figures and Tables

**Table 1:** Demographics and baseline characteristics of patients infected with 2019-nCoV

Clinical Characteristics	Patients (n=88)
**Age**	
Mean age (SD)	57.11 (15.39)
Range	21-89
Distribution no. (%)	
< 65	56 (63.64%)
≥ 65	32 (36.36%)
**Gender no. (%)**	
Male	36 (40.91%)
Female	52 (59.09%)
**BMI**	
Median (IQR)	24.12 (21.72-25.94)
Distribution no. (%)	
< 18.5	3 (3.41%)
18.5-24	34 (38.64%)
≥ 24	43 (48.86%)
**Onset of symptom to Hospital admission**	
Median days (IQR)	11.00 (6.50-14.50)
**Any comorbidity (%)**	41 (46.59%)
Hypertension	23 (26.14%)
Diabetes mellitus	11 (12.50%)
CAD	7 (7.95%)
COPD	4 (4.55%)
Malignancy	4 (4.55%)
Hyperthyroidism	2 (2.27%)
Depression	1 (1.14%)
Gallstones	1 (1.14%)
Gout	1 (1.14%)
Parkinson disease	1 (1.14%)
Cerebral infarction	1 (1.14%)
Kidney stones	1 (1.14%)
**Signs and symptoms (%)**	
Fever	63 (71.59%)
Cough	52 (59.09%)
Dyspnea	34 (38.64%)
Fatigue	26 (29.55%)
Diarrhea	22 (25.00%)
Myalgia	21 (23.86%)
Expectoration	15 (17.05%)
Chill	12 (13.64%)
Headache	12 (13.64%)
Pharyngalgia	5 (5.68%)
Nausea and vomiting	4 (4.55%)
Haemoptysis	2 (2.27%)
**Duration of 2019-nCoV nucleic acid**	
Median no. days (IQR)	22.00 (16.00-27.25)
**Outcome**	86 (97.73%)
Acute exacerbation	10 (11.36%)
Discharged	76 (86.36%)

Percentages do not total to 100%, owing to missing data; CAD, Coronary atherosclerotic heart disease. COPD, Chronic obstructive pulmonary disease.

**Table 2:** Laboratory findings of patients infected with 2019-nCoV on admission to hospital

Laboratory findings	Patients (n=88)
**WBC count (3.5-9.5×109/L)**	
Median (IQR)	5.41 (3.86-6.77)
Distribution no. (%)	
< 3.50	8 (9.09%)
3.50-9.50	52 (59.09%)
> 9.50	5 (5.68%)
**Lymphocyte count (1.1-3.2×109/L)**	
Median (IQR)	1.08 (0.71-1.50)
Distribution no. (%)	
< 1.10	44 (50.0%)
1.10-3.20	42 (47.73%)
**Eosinophil count (0.02-0.52×109/L)**	
Median (IQR)	0.01 (0.00-0.10)
Distribution no. (%)	
< 0.02	44 (50.0%)
0.02-0.52	40 (45.45%)
> 0.52	1 (1.14%)
**Monocyte count (0.10-0.60×109/L)**	
Median (IQR)	0.40 (0.32-0.56)
Distribution no. (%)	
0.10-0.60	70 (79.55%)
> 0.60	16 (18.18%)
**CRP (<1 mg/L)**	
Median (IQR)	14.70 (2.38-67.05)
Distribution no. (%)	
< 1.00	6 (6.82%)
≥ 1.00	59 (67.05%)
**ALT (≤ 33 U/L)**	
Median (IQR)	24.00 (15.00-47.00)
Distribution no. (%)	
≤ 33	56 (63.64%)
> 33	29 (32.95%)
**AST (≤ 32 U/L)**	
Median (IQR)	27.00 (20.00-44.00)
Distribution no. (%)	
≤ 32	33 (37.50%)
≥ 32	32 (36.36%)
**Globulin (20-35 g/L)**	
Median (IQR)	31.30 (29.35-34.65)
Distribution no. (%)	
20-35	66 (75.00%)
> 35	19 (21.59%)
**LDH (135-214 U/L)**	
Median (IQR)	251.0 (194.5-333.0)
Distribution no. (%)	
< 214	37 (42.05%)
≥ 214	48 (54.55%)
**Ferritin (15-150 ng/ml)**	
Median (IQR)	412.6 (158.4-952.2)
Distribution no. (%)	
< 150	14 (15.91%)
≥ 150	43 (48.86%)
**IL-1β (< 5.0 pg/ml)**	
Distribution no. (%)	
< 5.0	47 (53.41%)
≥ 5.0	5 (5.68%)
**IL-2R (223-710 U/ml)**	
Median (IQR)	437.5 (253.5-578.8)
Distribution no. (%)	
< 223	11 (12.50%)
223-710	34 (38.64%)
> 710	9 (10.23%)
**IL-6 (< 7.0 pg/ml)**	
Median (IQR)	3.02 (1.50-11.47)
Distribution no. (%)	
< 7	38 (43.18%)
≥ 7	15 (17.05%)
**IL-8 (< 62 pg/ml)**	
Median (IQR)	8.75 (5.00-13.48)
Distribution no. (%)	
< 62	48 (54.55%)
≥ 62	6 (6.82%)
**IL-10 (< 9.1 pg/ml)**	
Distribution no. (%)	
< 9.1	47 (53.41%)
≥ 9.1	4 (4.55%)
**TNF-α (< 8.1 pg/ml)**	
Median (IQR)	7.20 (5.70-7.20)
Distribution no. (%)	
< 8.1	25 (28.41%)
≥ 8.1	11 (12.50%)
**D-dimer (< 0.50 μg/ml)**	
Median (IQR)	0.61 (0.37-1.20)
Distribution no. (%)	
< 0.50	39 (44.32%)
≥ 0.50	40 (45.45%)
**PT (11.5-14.5 s)**	
Median (IQR)	13.90 (13.30-14.80)
Distribution no. (%)	
< 14.5	52 (59.09%)
≥ 14.5	26 (29.55%)
**cTnI (< 15.6 μg/L)**	
Median (IQR)	5.70 (2.80-11.50)
Distribution no. (%)	
< 15.6	52 (59.09%)
≥ 15.6	7 (7.95%)

Percentages do not total 100%, owing to missing data; WBC, White blood cell. CRP, C-reactive protein. ALT, Alanine aminotransferase. AST, Aspartate aminotransferase. LDH, Lactate dehydrogenase. IL, Interleukin. TNF, Tumor Necrosis Factor. PT, Prothrombin time. cTnI, cardiac troponin I.

**Table 3:** Demographics and baseline characteristics of patients infected with 2019-nCoV according to disease severity

Characteristics	Disease Severity			*p* value
	Moderate (n=47)	Severe (n=32)	Critically ill (n=9)	
**Age**				
Mean age (SD)	52.49 (14.62)	59.94 (13.96)	74.78 (10.06)	**< 0.001**
Distribution no. (%)				**< 0.001**
< 65	36/47 (76.60)	19/32 (59.38)	1/9 (11.11)	-
≥ 65	11/47 (23.40)	13/32 (40.62)	8/9 (88.89)	-
**Gender no. (%)**				**0.013**
Male	21/47 (44.68)	8/32 (25.00)	7/9 (77.78)	-
Female	26/47 (55.32)	24/32 (75.00)	2/9 (22.22)	-
**BMI**				
Median (IQR)	24.22 (21.75-25.89)	24.03 (22.04-27.35)	21.88 (16.24-25.45)	0.340
**Onset of symptom to Hospital admission**				
Median no. days (IQR)	11.00 (6.00-23.00)	11.00 (8.00-14.00)	13.00 (12.00-14.00)	0.710
**Any comorbidity (%)**	17/47 (36.17)	17/32 (53.13)	7/9 (77.78)	0.047
Hypertension	10 (58.82)	10 (58.82)	3 (42.86)	0.535
Diabetes mellitus	6 (35.29)	4 (23.53)	1 (14.29)	0.991
CAD	3 (17.65)	3 (17.65)	1 (14.29)	0.831
COPD	1 (5.88)	2 (11.76)	1 (14.29)	0.419
Malignancy	2 (11.76)	1 (5.88)	1 (14.29)	0.591
**Signs and symptoms (%)**				
Fever	32 (68.09)	24 (75.00)	7 (77.78)	0.728
Cough	31 (65.96)	18 (56.25)	3 (33.33)	0.174
Dyspnea	13 (27.66)	16 (50.00)	5 (55.56)	0.074
Fatigue	15 (31.91)	10 (31.25)	1 (11.11)	0.440
Diarrhea	7 (14.89)	13 (40.63)	2 (22.22)	0.034
**Duration of 2019- nCoV nucleic acid**				
Median no. days (IQR)	21.00 (15.50-24.50)	23.00 (18.75-28.00)	13.00 (5.00-29.00)	0.129
**Outcome**	47 (100.00)	31 (96.88)	8 (88.89)	<0.001
Discharged	46 (97.87)	30 (93.75)	0 (0)	
Acute exacerbation	1 (2.13)	1 (3.13)	8 (88.89)	

CAD, Coronary atherosclerotic heart disease. COPD, Chronic obstructive pulmonary disease.

**Table 4:** Laboratory findings of patients infected with 2019-nCoV on admission to hospital according to disease severity

Laboratory findings	Disease Severity			*p* value
	Moderate (n=47)	Severe (n=32)	Critically ill (n=9)	
**WBC count (3.5-9.5×10^9^/L)**				
Median (IQR)	5.06 (3.87-6.49)	5.24 (3.74-6.80)	6.15 (5.40-8.91)	0.174
Distribution no. (%)	27 (57.45)	31 (96.88)	7 (77.78)	0.489
< 3.50	4 (14.81)	4 (12.90)	0	-
3.50-9.50	19 (70.37)	26 (83.87)	7 (100.00)	-
> 9.50	4 (14.81)	1 (3.23)	0	-
**Lymphocyte count (1.1-3.2×10^9^/L)**				
Median (IQR)	1.34 (0.85-1.83)	0.89 (0.64-1.24)	0.57 (0.50-0.93)	**< 0.001**
Distribution no. (%)	47 (100.00)	32 (100.00)	7 (77.78)	**0.002**
< 1.10	16 (34.04)	22 (68.75)	6 (85.71)	-
1.10-3.20	31 (65.96)	10 (31.25)	1 (14.29)	-
**Eosinophil count (0.02-0.52×10^9^/L)**				
Median (IQR)	0.07 (0.01-0.15)	0.00 (0.00-0.02)	0.00 (0.00-0.02)	**< 0.001**
Distribution no. (%)	47 (100.00)	32 (100.00)	6 (66.67)	**0.002**
< 0.02	15 (31.91)	24 (75.00)	5 (83.33)	-
0.02-0.52	31 (65.96)	8 (25.00)	1 (16.67)	-
> 0.52	1 (2.13)	0	0	
**Monocyte count (0.10-0.60×10^9^/L)**				
Median (IQR)	0.41 (0.32-0.60)	0.39 (0.30-0.45)	0.36 (0.30-0.64)	0.508
Distribution no. (%)	47 (100.00)	32 (100.00)	7 (77.78)	0.226
0.10-0.60	36 (76.60)	29 (90.63)	5 (71.43)	-
> 0.60	11 (23.40)	3 (9.38)	2 (28.57)	-
**CRP (<1 mg/L)**				
Median (IQR)	7.20 (1.60-31.30)	33.20 (3.63-102.40)	98.60 (61.20-142.20)	**< 0.001**
Distribution no. (%)	27 (57.45)	31 (96.88)	7 (77.78)	0.347
<1.00	3 (11.11)	3 (9.68)	0	-
≥ 1.00	24 (88.89)	28 (90.32)	7 (100.00)	-
**ALT (≤ 33 U/L)**				
Median (IQR)	22.50 (14.00-46.50)	24.00 (16.00-38.00)	30.00 (16.00-54.00)	0.524
Distribution no. (%)	46 (97.87)	32 (100.00)	7 (77.78)	0.633
≤ 33	29 (63.04	23 (71.88)	4 (57.14)	-
> 33	17 (36.96)	9 (28.12)	3 (42.86)	-
**AST (≤ 32 U/L)**				
Median (IQR)	23.50 (17.75-34.25)	28.00 (21.00-54.50)	44.00 (41.00-56.00)	**0.002**
Distribution no. (%)	27 (57.45)	31 (96.88)	7 (77.78)	**0.002**
≤ 32	15 (55.56)	18 (58.06)	0	-
> 32	12 (44.44)	13 (41.94)	7 (100.00)	-
**Globulin (20-35 g/L)**				
Median (IQR)	30.70 (28.55-34.23)	32.65 (29.45-35.38)	33.70 (30.50-38.00)	0.242
Distribution no. (%)	46 (97.87)	32 (100.00)	7 (77.78)	0.290
20-35	38 (82.61)	24 (75.00)	4 (57.14)	-
> 35	8 (17.39)	8 (25.00)	3 (42.86)	-
**LDH (135-214 U/L)**				
Median (IQR)	201.5 (176.0-277.8)	297.0 (211.8-428.3)	493.0 (471.0-570.0)	**<0.001**
Distribution no. (%)	46 (97.87)	32 (100.00)	7 (77.78)	**< 0.001**
135-214	29 (63.04)	8 (25.00)	0	-
≥ 214	17 (36.96)	24 (75.00)	7 (100.00)	-
**Ferritin (15-150 ng/ml)**				
Median (IQR)	348.5 (136.3-590.9)	504.0 (148.3-956.0)	1853 (1222-3004)	**0.002**
Distribution no. (%)	33 (70.21)	19 (59.38)	5 (55.56)	0.409
< 150	9 (27.27)	5 (26.32)	0	-
≥ 150	24 (72.73)	14 (73.68)	5 (100.00)	-
**IL-1β (< 5.0 pg/ml)**				
Distribution no. (%)	27 (57.45)	18 (56.25)	7 (77.78)	0.7458
< 5.0	24 (88.89)	17 (94.44)	6 (85.71)	-
≥ 5.0	3 (11.11)	1 (5.56)	1 (14.29)	-
**IL-2R (223-710 U/ml)**				
Median (IQR)	297.0 (200.5-476.5)	478.0 (296.5-565.5)	968.0 (638.0-2279)	**0.005**
Distribution no. (%)	29 (61.70)	18 (56.25)	7 (77.78)	**< 0.001**
< 223	8 (27.59)	2 (11.11)	1 (14.29)	-
223-710	19 (65.52)	14 (77.78)	1 (14.29)	-
> 710	2 (6.89)	2 (11.11)	5 (71.42)	-
**IL-6 (< 7.0 pg/ml)**				
Median (IQR)	1.88 (1.50-5.76)	2.96 (1.89-14.07)	34.01 (8.71-158.10)	**0.002**
Distribution no. (%)	29 (61.70)	18 (56.25)	6 (66.67)	**0.005**
< 7	24 (82.76)	13 (72.22)	1 (16.67)	-
≥ 7	5 (17.24)	5 (27.78)	5 (83.33)	-
**IL-8 (< 62 pg/ml)**				
Median (IQR)	7.10 (5.00-10.60)	9.25 (7.25-12.73)	76.10 (18.80-436.00)	**0.003**
Distribution no. (%)	29 (61.70)	18 (56.25)	7 (77.78)	**< 0.001**
< 62	28 (96.55)	17 (94.44)	3 (42.86)	-
≥ 62	1 (3.45)	1 (5.56)	4 (57.14)	-
**IL-10 (< 9.1 pg/ml)**				
Distribution no. (%)	27 (57.45)	18 (56.25)	6 (66.67)	**0.046**
< 9.1	26 (96.30)	17 (94.44)	4 (66.67)	-
≥ 9.1	1 (3.70)	1 (5.56)	2 (33.33)	-
**TNF-α (< 8.1 pg/ml)**				
Median (IQR)	6.80 (5.40-7.60)	6.60 (5.10-10.50)	11.50 (8.50-22.50)	**0.008**
Distribution no. (%)	12 (25.53)	17 (53.13)	7 (77.78)	**0.008**
< 8.1	12 (100.00)	11 (64.71)	2 (28.57)	-
≥ 8.1	0	6 (35.29)	5 (71.43)	-
**D-dimer (< 0.50 μg/ml)**				
Median (IQR)	0.49 (0.32-0.81)	0.70 (0.40-1.57)	0.83 (0.65-6.64)	0.086
Distribution no. (%)	42 (89.36)	30 (93.75)	7 (77.78)	**0.028**
< 0.50	26 (61.90)	12 (40.00)	1 (14.29)	-
≥ 0.50	16 (38.10)	18 (60.00)	6 (85.71)	-
**PT (11.5-14.5 s)**				
Median (IQR)	13.65 (13.18-14.53)	14.20 (13.60-15.20)	15.80 (13.20-16.20)	**0.019**
Distribution no. (%)	42 (89.36)	29 (90.63)	7 (77.78)	0.062
< 14.5	31 (73.81)	19 (65.52)	2 (28.57)	-
≥ 14.5	11 (26.19)	10 (34.48)	5 (71.43)	-
**cTn I (< 15.6 μg/L)**				
Median (IQR)	3.30 (2.63-5.65)	6.30 (3.23-12.55)	8.70 (6.30-70.70)	**0.008**
Distribution no. (%)	31 (65.96)	21 (65.63)	7 (77.78)	**0.025**
< 15.6	29 (93.55)	19 (90.48)	4 (57.14)	-
≥ 15.6	2 (6.45)	2 (9.52)	3 (42.86)	-

WBC, White blood cell. CRP, C-reactive protein. ALT, Alanine aminotransferase. AST, Aspartate aminotransferase. LDH, Lactate dehydrogenase. IL, Interleukin. TNF, Tumor Necrosis Factor. PT, Prothrombin time. cTnI, cardiac troponin I.

**Table 5:** The relations between different symptoms and inflammatory indexes

	Dyspnea		*p*	Fever		*p*
	Yes (n=34)	No (n=54)		Yes (n=63)	No (n=25)	
**Lymphocyte count (1.1-3.2×10^9^/L)**						
Median (IQR)	0.84 (0.54-1.34)	1.21 (0.85-1.67)	**0.008**	0.93 (0.68-1.35)	1.33 (0.90-1.81)	**0.049**
**Eosinophil count (0.02-0.52×10^9^/L)**						
Median (IQR)	0.01 (0.00-0.08)	0.02 (0.00-0.14)	0.126	0.01 (0.00-0.09)	0.07 (0.01-0.15)	**0.021**
**CRP (< 1 mg/L)**						
Median (IQR)	34.50 (5.00-90.35)	10.90 (1.70-48.65)	**0.036**	28.85 (3.68-81.28)	5.40 (1.10-34.65)	**0.031**
**IL-6 (< 7.0 pg/ml)**						
Median (IQR)	6.67 (2.25-26.81)	2.04 (1.50-5.76)	**0.015**	5.04 (1.60-25.36)	2.26 (1.50-5.13)	0.064
**IL-8 (< 62 pg/ml)**						
Median (IQR)	9.60 (7.25-54.20)	7.10 (5.00-12.70)	0.109	9.90 (5.38-20.00)	7.10 (5.00-9.63)	**0.045**
**TNF-α (< 8.1pg/ml)**						
Median (IQR)	7.90 (5.50-11.20)	5.80 (4.60-7.35)	**0.036**	6.90 (4.63-10.25)	6.05 (5.25-7.50)	0.436
**PT (11.5-14.5 s)**						
Median (IQR)	14.50 13.68-15.88)	13.70 (13.03-14.38)	**<0.001**	13.90 (13.23-15.00)	13.85 (13.30-14.43)	0.591

CRP, C-reactive protein. IL, Interleukin. TNF, Tumor Necrosis Factor. PT, Prothrombin time.

**Table 6:** Analysis of factors influencing the prognosis of patients infected with 2019-nCoV

Factors	Discharged (n=76)	Acute exacerbation (n=10)	*p*
**Gender**	27	8	**0.013**
Male	27	8	
Female	49	2	
**Age**			
< 65	52	3	**0.031**
≥ 65	24	7	
**Disease severity**			
Mild	46	1	**<0.001**
Severe	30	1	
Critically ill	0	8	
**BMI**			
<18.5	2	1	0.058
18.5-24	30	2	
>24	42	1	
**Any comorbidity**			
Yes	33	7	**0.014**
No	33	0	
**Glucocorticoid use**			
Yes	26	6	0.164
No	50	4	
**WBC count (×10^9^/L)**			
<3.5	9	1	0.820
3.5-9.5	62	6	
>9.5	5	1	
**Lymphocyte count (×10^9^/L)**			
<1.1	35	7	0.057
1.1-3.2	41	1	
**Eosinophil count (×10^9^/L)**			
<0.02	36	6	0.151
0.02-0.52	39	1	
>0.52	1	0	
**Monocyte count (×10^9^/L)**			
0.1-0.6	62	6	0.644
>0.6	14	2	
**CRP (mg/L)**			
<1.0	10	1	>0.990
≥1.0	66	7	
**ALT (U/L)**			
<33	50	6	>0.990
≥33	25	2	
**AST (U/L)**			
<32	51	0	**<0.001**
≥32	24	8	
**Globulin (g/L)**			
20-35	59	6	>0.990
>35	16	2	
**LDH (U/L)**			
<214	37	0	**0.008**
≥214	38	8	
**Ferritin (ng/ml)**			
<150	14	0	0.319
≥150	38	5	
**IL-1β (pg/ml)**			
<5.0	47	6	0.548
≥5.0	5	1	
**IL-6 (pg/ml)**			
<7.0	41	1	**0.005**
≥7.0	11	5	
**IL-2R (U/ml)**			
<223	12	1	**<0.001**
223-710	37	0	
>710	3	6	
**IL-8 (pg/ml)**			
<62	50	4	**0.010**
≥62	2	3	
**IL-10 (pg/ml)**			
<9.1	48	4	0.053
≥9.1	2	2	
**TNF-α (pg/ml)**			
<8.1	41	3	0.051
≥8.1	10	4	
**D-dimer (μg/ml)**			
<0.5	39	1	**0.027**
≥0.5	31	7	
**PT (s)**			
<14.5	49	3	0.104
≥14.5	20	5	
**cTnI (μg/L)**			
<15.6	48	3	**0.019**
≥15.6	4	3	

## Abbreviations

COVID-19: novel coronavirus disease 2019
BMI: body mass index
CAD: coronary atherosclerotic heart disease
2019-nCoV: 2019 novel coronavirus
SARS: severe acute respiratory syndrome
MERS: Middle East respiratory syndrome
ICU: intensive care unit
ESR: Erythrocyte sedimentation rate
CRP: C-reactive protein
CT: computed tomography
RT-PCR: real time polymerase chain reaction
ALT: alanine aminotransferase
AST: aspartate aminotransferase
IL: Interleukin
TNF-α: Tumor Necrosis Factor α
SD: standard deviation
IQR: interquartile range
DM: diabetes mellitus
COPD: chronic obstructive pulmonary disease
WBC: white blood cell count
PT: prothrombin time
Th1: T-helper-1
Th2: T-helper-2
SARS-CoV: Severe Acute Respiratory Syndrome Coronavirus
MERS-CoV: Middle East Respiratory Syndrome Coronavirus
